# When Treatment Becomes a Threat: A Report of a Rare Case of 5-Fluorouracil (5-FU)-Induced Enteritis Mimicking Inflammatory Bowel Disease

**DOI:** 10.7759/cureus.111505

**Published:** 2026-06-25

**Authors:** Andreas Sinanan, Shiva Verma, Yao X Schmidt, Parmod Kumar

**Affiliations:** 1 Internal Medicine, Sierra View Medical Center, Porterville, USA; 2 Pathology, Sierra View Medical Center, Porterville, USA; 3 Gastroenterology, Sierra View Medical Center, Porterville, USA

**Keywords:** 5-fu toxicity, ibd, leucovorin, non-ibd colitis, severe enteritis

## Abstract

We report a rare case of 5-fluorouracil-induced enteritis mimicking inflammatory bowel disease (IBD). A 67-year-old man undergoing adjuvant chemotherapy for colon cancer developed profuse diarrhea and fever. Initial findings were suggestive of IBD; however, biopsy findings and clinicopathologic correlation supported a diagnosis of chemotherapy-induced enteritis. Cessation of chemotherapy and initiation of corticosteroids led to resolution of symptoms. Awareness of this rare complication can prevent misdiagnosis and inappropriate treatment.

## Introduction

5-Fluorouracil (5-FU) is a core component of adjuvant chemotherapy for colorectal carcinoma but is frequently associated with GI toxicity, most commonly diarrhea [1-3]. Severe small bowel enteritis, however, is rare and underreported in the literature [1,2,4,5]. Leucovorin potentiates 5-FU cytotoxicity through stabilization of the fluorodeoxyuridylate-thymidylate synthase ternary complex, enhancing both antitumor activity and GI mucosal injury [3]. The resulting mucosal damage can closely resemble inflammatory bowel disease (IBD), both clinically and histologically, posing diagnostic challenges that may lead to unnecessary immunosuppression [1,2]. We describe a case of 5-FU-induced enteritis mimicking IBD in a patient receiving adjuvant chemotherapy for colon cancer.

## Case presentation

A 67-year-old man with stage IIA transverse colon adenocarcinoma underwent partial colectomy with primary anastomosis. He was started on adjuvant chemotherapy with 5-FU (500 mg/m²) and leucovorin (500 mg/m²) per standard regimens [2].

After the first treatment cycle, he developed fever and more than 40 episodes of watery diarrhea over 48 hours. Physical examination showed mild diffuse abdominal tenderness. Laboratory evaluation revealed an elevated fecal calprotectin level of 3340 μg/g. Comprehensive stool studies, including *Clostridioides difficile *testing, were negative.

**Figure 1:**
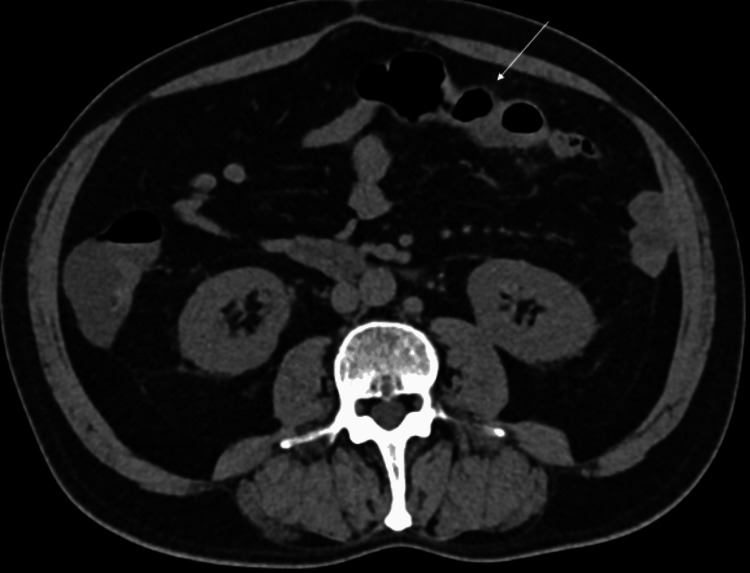
Contrast-enhanced CT of the abdomen and pelvis showing mild thickening of the transverse colon

**Figure 2:**
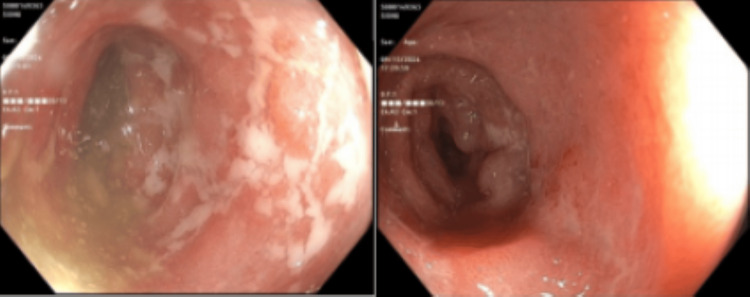
Colonoscopic images demonstrating ulcerations in the terminal ileum with surrounding erythema, initially suggestive of IBD IBD, inflammatory bowel disease

**Figure 3:**
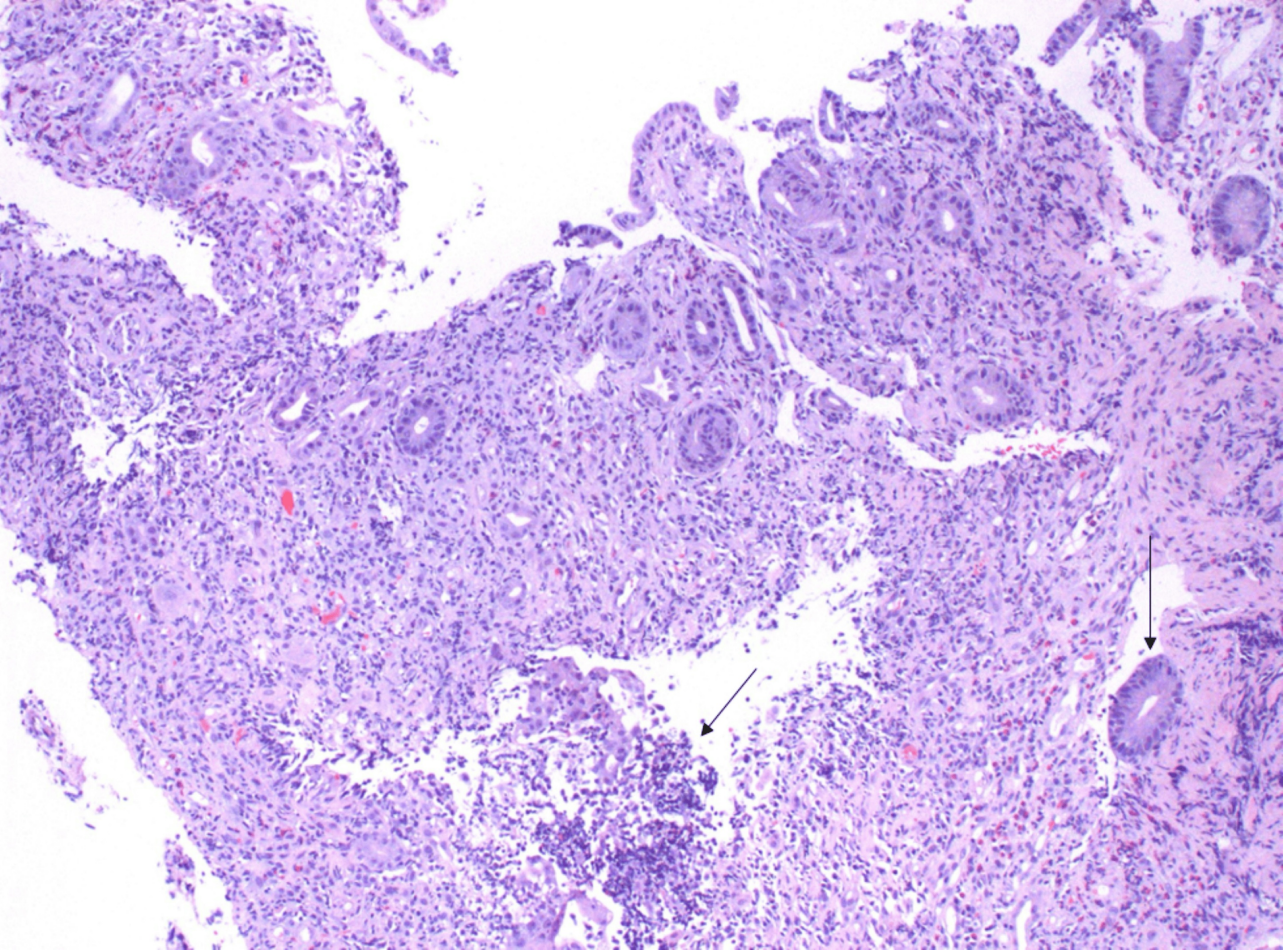
Endoscopic biopsy of the terminal ileum revealing ulceration, distortion of glandular architecture, and eosinophilic infiltration

Contrast-enhanced CT of the abdomen and pelvis demonstrated mild thickening of the transverse colon (Figure 1). Colonoscopy on hospital day 6 showed erythematous mucosa and multiple ulcerations in the terminal ileum (Figure 2). Biopsy of the ileal ulcers revealed acute enteritis with epithelial injury, architectural distortion, and eosinophilic infiltration (Figure 3). These findings aligned with published reports of chemotherapy-induced small bowel toxicity with features that may mimic IBD [1,2].

A timeline of clinical events is presented in Table 1. 

**Table 1:** Timeline of clinical events 5-FU, 5-fluorouracil

Time point	Clinical event
Prehospital	Initiation of the first cycle of 5-FU 500 mg/m² and leucovorin 500 mg/m²
Day 1	Week 7 of 5-FU and leucovorin chemotherapy
Day 1	Hospital admission for >48 hours of watery diarrhea; empiric antibiotics were started for suspected colitis based on CT findings.
Day 6	Colonoscopy showed ulcerations in the terminal ileum.
Day 6	Initiation of sulfasalazine 500 mg PO twice daily, folic acid 1 mg PO daily, methylprednisolone 40 mg IV every 12 hours, and azathioprine 50 mg PO twice daily
Day 7	Complete resolution of diarrhea
Post-endoscopic evaluation	Literature review identified six previously reported cases of 5-FU- and leucovorin-induced enteritis.
Day 8	Sulfasalazine and azathioprine were discontinued; the patient was discharged on a three-week prednisone taper.
Outpatient oncology follow-up	5-FU and leucovorin were discontinued; the patient was transitioned to capecitabine 1,500 mg PO twice daily in two-week cycles with one-week breaks (20-week regimen).
Subsequent course	Good clinical response; reduced-dose rechallenge with 5-FU and leucovorin was not attempted.

Initially, the patient received azathioprine, sulfasalazine, and IV corticosteroids for presumed IBD. However, clinicopathologic correlation and a review of the literature supported a diagnosis of fluorouracil-induced enteritis potentiated by leucovorin [2,3]. Immunomodulators were discontinued, and corticosteroids were transitioned to an oral taper; diarrhea resolved within 24 hours.

The 5-FU/leucovorin regimen was discontinued. The patient was transitioned to oral capecitabine for 20 weeks. He tolerated this regimen without recurrence of GI symptoms.

## Discussion

This case highlights a rare manifestation of chemotherapy-related GI toxicity: 5-FU-induced enteritis in a patient receiving adjuvant 5-FU and leucovorin. The patient presented with acute-onset profuse diarrhea, fever, and terminal ileal ulcerations on colonoscopy, findings that initially raised concern for IBD. However, the sudden onset of symptoms, close temporal relationship to chemotherapy administration, and the patient's age at presentation were atypical for new-onset IBD, prompting consideration of an alternative diagnosis [1].

Diarrhea is a well-recognized adverse effect of fluoropyrimidine-based chemotherapy and is thought to result from injury to rapidly dividing GI epithelial cells [3-5]. 5-FU exerts its antineoplastic effect primarily through inhibition of thymidylate synthase, disrupting DNA synthesis and ultimately leading to cellular apoptosis [3,5]. Although GI toxicities such as mucositis, nausea, vomiting, and diarrhea are frequently encountered, severe enteritis involving the small bowel has only rarely been described in the literature [1,2]. When present, the resulting inflammation can produce endoscopic and histologic findings that closely resemble infectious enteritis or IBD, making diagnosis particularly challenging [1,2,4].

The concomitant use of leucovorin may further contribute to the severity of GI toxicity. Leucovorin enhances the activity of 5-FU by stabilizing its binding to thymidylate synthase, thereby increasing both antitumor efficacy and the potential for mucosal injury [3]. This mechanism has been proposed as a contributing factor in previously reported cases of severe enteritis associated with combination therapy [2].

Recognition of this uncommon adverse effect is important, as delayed diagnosis may result in unnecessary investigations, inappropriate treatment, or continued exposure to the causative agent. In our patient, symptoms improved rapidly following discontinuation of chemotherapy and initiation of IV methylprednisolone. Clinical improvement was evident within 24 hours, and he subsequently completed a three-week prednisone taper with complete resolution of symptoms.

Reports of 5-FU-induced enteritis remain scarce. In the largest published series, six patients developed acute small bowel toxicity after treatment with 5-FU and leucovorin [1]. Five patients subsequently underwent rechallenge; one experienced recurrent toxicity, whereas four tolerated further treatment, generally with dose modification [1]. Our patient elected not to undergo rechallenge and was transitioned to oral capecitabine, completing a 20-week course without further GI complications. Although capecitabine is a prodrug of 5-FU and may also cause GI toxicity, successful use following 5-FU-associated enteritis has been reported and was well tolerated in our patient [2,6].

To our knowledge, this case represents the eighth reported case of 5-FU-associated enteritis. Awareness of this entity is essential, particularly in oncology patients presenting with severe diarrhea and inflammatory changes on endoscopy shortly after chemotherapy exposure. Early recognition and prompt withdrawal of the offending agent can help avoid diagnostic confusion, prevent unnecessary treatment, and improve patient outcomes.

## Conclusions

5-FU-induced enteritis should be included in the differential diagnosis in any patient receiving chemotherapy who presents with acute GI symptoms. Prompt recognition and withdrawal of the offending agent, along with supportive therapy, can lead to rapid symptom resolution and avoid inappropriate interventions.
